# Supplementary material for: Resistin Enhances VCAM-1 Expression and Monocyte Adhesion in Human Osteoarthritis Synovial Fibroblasts by Inhibiting MiR-381 Expression through the PKC, p38, and JNK Signaling Pathways
Source: Cells. 2020 Jun 1;9(6):1369. doi: 10.3390/cells9061369 (PMC7349127; doi:10.3390/cells9061369)
Supplement: Supplementary file 1 [file cells-09-01369-s001.pdf]

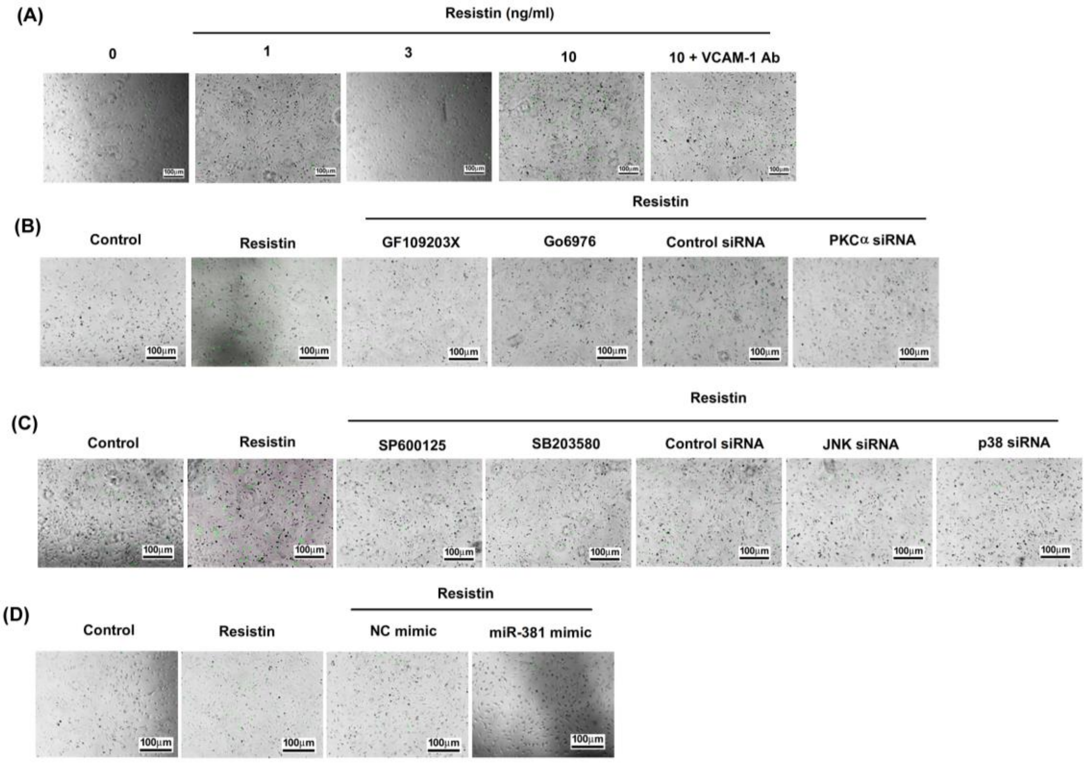


**Figure 1.** Resistin-enhanced VCAM-1 expression and monocyte adhesion by inhibiting miR-381 synthesis via the PKC, p38, and JNK signaling pathways. (**A**) OASFs were incubated with resistin (0, 1, 3, and 10 ng/mL) only or resistin 10 ng/mL + VCAM-1 antibody for 24 h. (**B**–**D**) OASFs were pretreated with a PKC inhibitor (GF109203x), a specific PKC$\alpha$/β inhibitor (Gö6976), JNK (SP600125) and p38 (SB203580), or transfected with PKC$\alpha$, JNK,p38, miR-381 mimic, NC mimic and control siRNA, then incubated with resistin for 24 h. Adherence of THP-1 cells to cultured OASFs was photographed by fluorescence microscopy, THP-1 cells were subsequently added to OASFs for 1 h. Adherence of THP-1 cells to cultured OASFs was photographed by fluorescence microscopy and quantified ($n$ = 4).

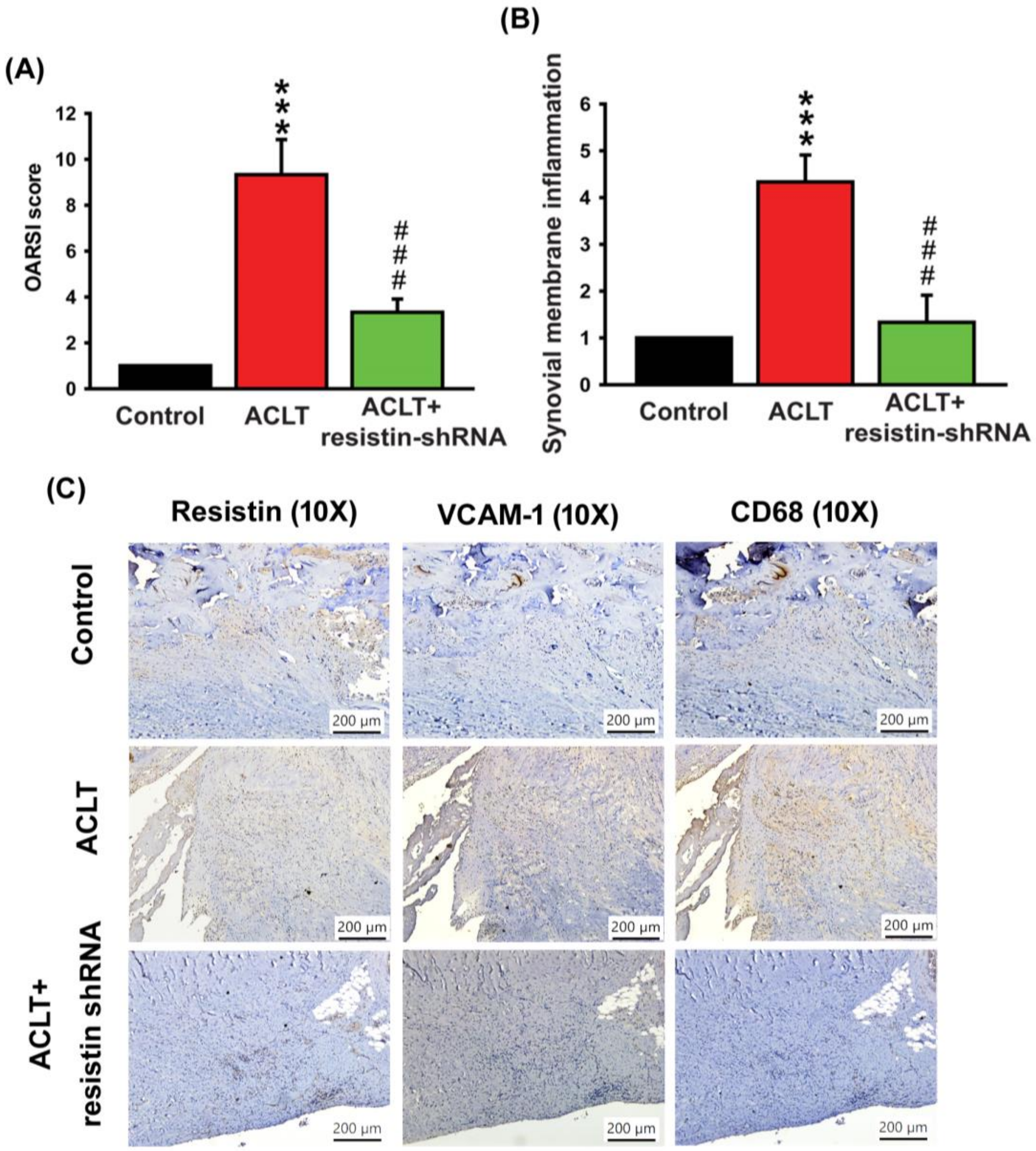


**Figure 2.** Resistin shRNA ameliorates histologic severity of OA. (**A**) OARSI score. (**B**) synovitis membrane inflammation. (**C**) Specimens from the control knee, ACLT knee, and resistin-shRNA-transfected ACLT knee were immunostained with resistin, VCAM-1, and CD68 (monocyte surface marker). * $p < 0.05$; ** $p < 0.01$; *** $p < 0.001$ compared with control knees; # $p < 0.05$; ## $p < 0.01$; ### $p < 0.001$ compared with the ACLT group.
